# Normal fault earthquakes or graviquakes

**DOI:** 10.1038/srep12110

**Published:** 2015-07-14

**Authors:** C. Doglioni, E. Carminati, P. Petricca, F. Riguzzi

**Affiliations:** 1 Dipartimento di Scienze della Terra, Università Sapienza, Roma, Italy; 2 Istituto di Geologia Ambientale e Geoingegneria, CNR, Roma, Italy; 3 GFZ-German Research Centre for Geosciences, Potsdam; 4 Istituto Nazionale di Geofisica e Vulcanologia, Roma, Italy

## Abstract

Earthquakes are dissipation of energy throughout elastic waves. Canonically is the elastic energy accumulated during the interseismic period. However, in crustal extensional settings, gravity is the main energy source for hangingwall fault collapsing. Gravitational potential is about 100 times larger than the observed magnitude, far more than enough to explain the earthquake. Therefore, normal faults have a different mechanism of energy accumulation and dissipation (graviquakes) with respect to other tectonic settings (strike-slip and contractional), where elastic energy allows motion even against gravity. The bigger the involved volume, the larger is their magnitude. The steeper the normal fault, the larger is the vertical displacement and the larger is the seismic energy released. Normal faults activate preferentially at about 60° but they can be shallower in low friction rocks. In low static friction rocks, the fault may partly creep dissipating gravitational energy without releasing great amount of seismic energy. The maximum volume involved by graviquakes is smaller than the other tectonic settings, being the activated fault at most about three times the hypocentre depth, explaining their higher b-value and the lower magnitude of the largest recorded events. Having different phenomenology, graviquakes show peculiar precursors.

Earthquakes dissipate energy stored by pressure gradients at plate boundaries and still represent a major issue both for public safety and for their mechanisms^1^,^2^. We assume that the lateral variations of the viscous-plastic basal mantle drag are determining the tectonics at plate boundaries and the deformation is transferred from the lithosphere base at the Earth’s surface. Due to the brittle nature of the upper crust, the shallow deformation occurs episodically, i.e., releasing in few seconds the energy accumulated in hundreds of years. We focus our study on the earthquakes generated by normal faults, which are widespread in several geodynamic environments, such as continental rifts, backarc basins, mid-ocean ridges, orogens and strike-slip settings. Earthquakes modify Earth’s gravitational energy^3^,^4^. It was pointed out that the coseismic gravitational energy variation might be few orders of magnitude larger than the radiated seismic wave energy, which is usually referred as ‘seismic energy’^5^,^6^. Unlike thrust or reverse faults^7^, gravity favours normal faulting since the maximum stress axis is parallel to the lithostatic load. In fact, contrary to thrust faults, normal fault rupture tends to propagate upward^8^. In extensional environments, the differential stress necessary to generate rock failure is on average 5–6 times smaller than that required in contractional tectonic settings. Accordingly, normal fault-related earthquakes never reached the magnitudes (e.g., >Mw 8.5) recorded in strike-slip and contractional settings and have a higher *b*-value of the Gutenberg-Richter power law^9^. The elastic rebound is commonly considered as the main model for earthquake generation^2^, being inferred as the mechanism dissipating the elastic energy accumulated during the interseismic period^2^. This is likely true for contractional and strike-slip tectonic settings, but in tensional environments, the influence of gravitation may rather be dominant^10^,^11^,^12^. In this paper we discuss some basic parameters that control the energy dissipation in shallow crustal extensional settings, such as the involved volume, the dip of the normal fault and the static friction^13^,^14^. Natural examples will be taken from the Apennines belt and other geodynamic settings, characterized by widespread extensional fault activity and related earthquakes. In addition we address the energy partitioning of earthquakes^15^ comparing the potential energy stored by the volume involved during the coseismic collapse^11^ with that inferred from earthquake magnitude. Regardless its origin (elastic or gravitational), potential energy has been demonstrated far greater, indicating that, in the energy budget, the available energy is far larger than that released by earthquakes waves. Therefore most of the energy must be dissipated by other geological phenomena (shear heating, heat flow and fracturing above all), consistently with previous works^5^,^16^. It was also shown that all earthquakes gradually decrease the global gravitational energy, which is transformed into heat flow^4^. However, as intuitively expected, in extensional tectonic settings the gravitational potential energy is decreasing, whereas it is increasing in contractional tectonic environments^3^.

In an upper crust having average density of 2.5 g/cm^3^, the vertical (lithostatic) load increases by about 25 MPa/km. Below 1–1.5 km depth, the crust is under horizontal compression even in extensional settings, i.e., the sigma 1 is vertical and corresponds to the lithostatic load, whereas the sigma 2 and 3 stress axes are horizontal but still contracting rocks. Therefore, say at 10 km depth, both sigma 2 and 3 must be positive and lower than 250 MPa. With the progression of the stretching, sigma 2 and 3 decrease, providing a larger differential stress that may eventually evolve into rupture and fault activation. This determines the collapse of the normal fault hangingwall, dissipating mostly gravitational potential energy^17^,^10^,^11^,^12^, being the elastic component a minor factor in the fall, if any.

## Geological model

**Figure 1:**
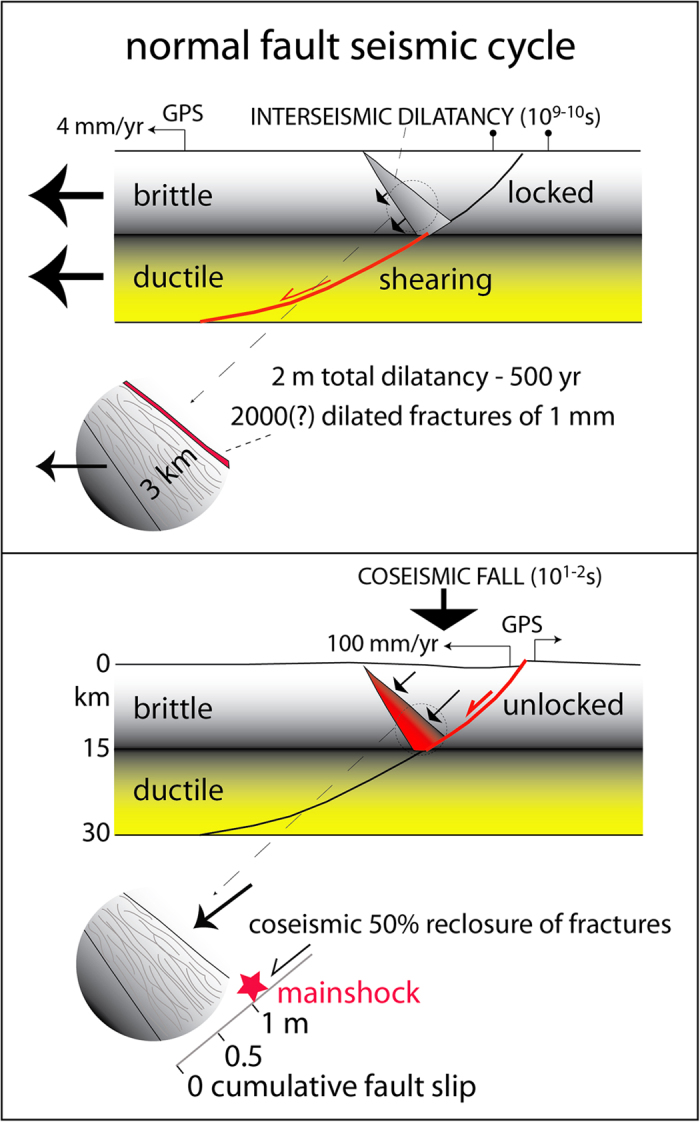
Geological model of the seismic cycle. During the interseismic period, while the lower crust shears steadily, the brittle upper crust is locked and a dilating wedge is inferred. The width of this triangle is here hypothetically imaged to affect an antithetic section to the locked fault, say 3 km thick. Partial sealing of the fractures due to fluids circulation may be expected. The remaining open fractures allow the fall of the hangingwall at the coseismic stage, when the fault plane and the dilated wedge cannot sustain anymore the upper suspended block. The coseismic collapse of the hangingwall could recover for example only half of the total extension. Note that the mainshock is located along the fault at the upper tip of the dilated wedge, consistently with the seismological observations showing that the mainshock is located slightly above the rupture zone deeper tip (e.g., like in the L’Aquila, 6 April 6.3 Mw earthquake).

In a simplified two-layers stretching crust, within the brittle upper crust, the faults are generally locked or slowly creeping, and the deformation is mostly assumed to be stick-slip. During the secular interseismic period of lithospheric stretching, the ductile lower crust is permanently shearing and thinning by viscous flow and deformation is inferred as a continuous process^10^. The brittle-ductile transition (BDT) is on average located in the middle Earth’s crust. When a brittle fault merges into a ductile shear zone crosscutting the whole crust, the BDT is characterized by a pressure gradient because the brittle upper crust is mostly locked, whereas the viscous-plastic lower crust is sheared steadily (Fig. 1).

Since the steady deformation of the ductile lower crust has to be transferred upward, it was proposed^10^ that the stretching could be accommodated in the brittle realm by dilation in a wedge conjugate to the episodically active normal fault (Fig. 1). In that wedge, millimetric open fractures are inferred to develop. These fractures may be partly filled by cement, and partly by fluids, as shown in logs of hydrocarbon exploration boreholes and as predicted by analogue modelling^18^. This mechanism has also been defined as dilatancy, i.e., the phenomenon in which fractures and cracks form and open when rocks are stressed^19^,^20^. This dilated wedge is inferred due to the strain partitioning and the pressure gradient between the ductile lower crust and the brittle upper crust. The occurrence of the dilated wedge from the geological model is also supported by the fact that the hangingwall of a normal fault could not collapse without a corresponding vacuity at the base of the activated fault segment (Fig. 1). A 10–15 km thick brittle crust needs only about 100–150 MPa to fail under extension. Moreover, once rocks are broken (e.g., by fracturing in limestone), they lose their elastic component, and fractures may be filled or unfilled by cement (Fig. 1), depending on fluids circulation, carbonate compensation depth, temperature, pressure, CO_2_ content in the system, etc. During the initial stage of collapse, fluid pressure increases^21^,^22^, supporting the existence, in pre-coseismic stages, of open fractures filled by fluids, becoming squeezed by the fall of the fault hangingwall.

In the hypothetical case the upper crust was made of low strength material, the deformation in the upper crust would rather occur in steady state, without generation of pressure gradients. In this situation, in the low-temperature upper crust, the fault would continuously creep and the conjugate dilatational wedge of Fig. 1 would not form. Therefore, the inferred dilated conjugate wedge reaches its maximum expression when the fault in the brittle crust is completely locked. Intermediate cases should possibly exist between these two end-members, and it is evident that the crust consists of multilayers having strength variability, hence generating multiple stress gradients.

The opening of fractures and the fluids permeating them gradually weaken the dilated wedge, which should increasingly lose strength during the interseismic stage. Therefore, the suspended hangingwall is lying on the fault on one side, and is bounded on the other side by the dilated wedge. When the fault and the conjugate wedge will not be any more sufficiently strong to support the hangingwall, the sudden collapse will generate the earthquake (Fig. 1). Therefore, from the BDT to the surface there may be accumulation of elastic and gravitational potential energy in a “suspended” volume^10^,^11^. The volume times its density gives the mass of the hangingwall wedge. The hangingwall will collapse when the weight of this volume will overtake the strength of the fault plane and of the dilated wedge. At the coseismic stage the wedge will partly recover, by fracture closure, the dilation accommodated during the interseismic period (Fig. 1). This is proposed to be accompanied by expulsion of fluids that permeated the fractures^11^.

Alternatively, the fall of the hangingwall may be diluted in time during the interseismic stage generating continuous microseismicity in case of lower fault dip^23^, possibly associated with lower friction. The Mw 6.3 2009 April 6^th^ L’Aquila earthquake can be taken as a case history for this model. During the 4–5 months preceding the quake, a series of foreshocks occurred in the volume of the hangingwall and along the inferred dilated wedge^10^.

The model predicts that the dilated wedge formed during the interseismic will be contracted during the coseismic stage. An example of this inversion occurred during the 1986 Kalamata (South Peloponnesus) normal fault-related earthquake, which was associated with several compressional focal mechanisms^24^. The opposite behaviour of interseismic contraction and coseismic extension is predicted for thrust faults^10^; one example is the normal fault earthquake associated to the 2011 Tohoku megathrust^25^.

**Figure 2:**
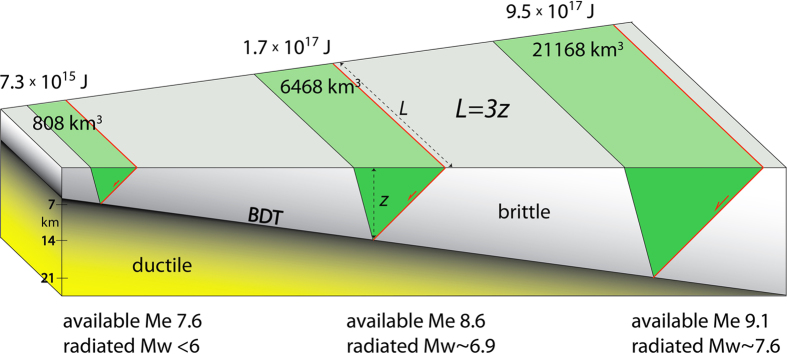
Relationship between normal fault length, brittle-ductile transition (BDT) depth, and maximum involved hangingwall volume. Three cases of 45° dipping normal faults, with BDT and hypocentre depths at 7, 14 and 21 km, with 0.5, 1.5 and 3 m of coseismic slip respectively, with a vertical component of 0.35, 1.06, and 2.12 m. The volume is computed assuming that the length (L) of the activated fault along strike is three times the hypocentre depth (z). The inferred conjugate boundary of the normal fault limiting the volume is about 60° and represents the inferred dilated wedge during the interseismic period. Doubling the depth of the hypocentre, the volume increases 8 times. Triplicating the depth the volume increases 26 times. The maximum potential energy in joule of the three suspended volumes above a normal fault increases of about one order of magnitude for each deepening of the BDT. Normal fault earthquakes are predicted to reach maximum magnitude when hypocentres are located close to the BDT (being the BDT deeper where the surface heat flow is lower) and rupture propagates up to the surface. Therefore, the deeper the BDT, the larger the volume and the higher the earthquake magnitude. Me, is the potential magnitude gravitational energy computed for the green volume; Mw, is the magnitude momentum, i.e., the seismic energy released during the real quake as instrumentally measured, being about 1.5 - 1.7 lower. Therefore, in terms of energy ratios, only about 1% of the potential gravitational energy is transformed into seismic energy.

Fault rupture length increases with earthquake magnitude and depends also on the tectonic setting^26^. Based on the deformed volume constrained by seismic sequences in Italy^27^,^28^, regional studies of surface ruptures^29^ and InSAR and GPS data^30^,^31^, normal fault-related seismicity is usually associated with rupture fault length about three times the hypocentre depth (Fig. 2). InSAR data indicate that epicentres are regularly located at the margin of the collapsed volume opposite to the seismogenic fault, where the coloured cycles of the interferogram fringes decrease or disappear^31^. In the volume surrounding the collapsed hangingwall, coseismic uplift is frequently observed, consistent with a rebound to the gravitational collapse. Therefore, assuming a dip of about 45° of the wedge conjugate to a fault activated during an earthquake nucleated near the BDT, the volume of the wedge falling at the coseismic stage can be calculated (Fig. 2). In addition, it is well known that fault length is correlated to fault displacement^32^. This means that that the deeper the BDT in a given period of crustal evolution, the longer the associated faults. Moreover this implies that the bigger the related volume and the larger the displacement and the dissipated energy.

## Normal fault dip and friction

**Figure 3:**
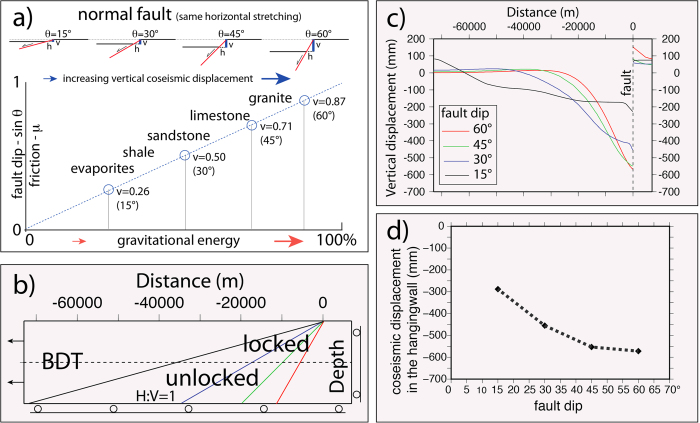
Fault dip, friction, gravitational potential energy and numerical modelling. **a**) For a given amount of crustal extension, which is represented by the horizontal component of the normal fault displacement (h), the dip of the normal fault (ϑ) controls the vertical displacement (v). The shallower the dip, the smaller the vertical displacement. Therefore, steeper faults release larger amounts of gravitational potential energy at the earthquake. This is consistent with the absence of energetic earthquakes associated with low-angle normal faults. It is observed that low-angle normal faults develop in rocks having low friction and vice-versa steep normal faults form in high friction rocks. (**b–d**) Finite elements numerical modelling was performed to evaluate vertical displacement and coseismic slip as a function of the normal fault dip. (**b**) Model setup: the model is 20 km thick, and the brittle ductile transition (BDT) is fixed at 10 km depth. The crust is gradually stretched of 1 m. Above the BDT, faults are locked during the interseismic period, and unlocked in the lower crust. (**c**) During the coseismic stage, the brittle segment of the fault is unlocked and the vertical displacement is predicted to be a function of the fault dip. (**d**) The maximum hangingwall coseismic slip (at the fault) increases with the fault dip, supporting the model of (**a**) (see text).

**Figure 4:**
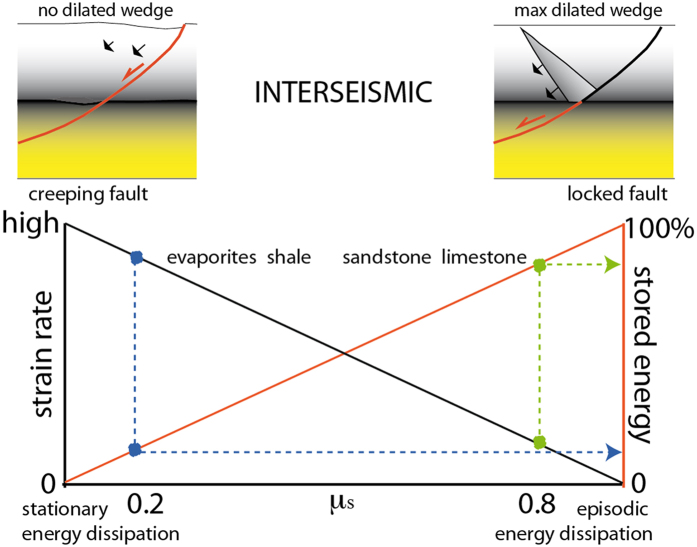
Relationship between strain rate (SR), friction and stored potential energy. SR decreases with friction and in fact the most energetic earthquakes occur in active tectonic zones where the strain rate is relatively lower. This implies that low friction allows creeping and more continuous dissipation of energy, whereas high friction determines locked faults and storage of larger energy during the interseismic period.

**Figure 5:**
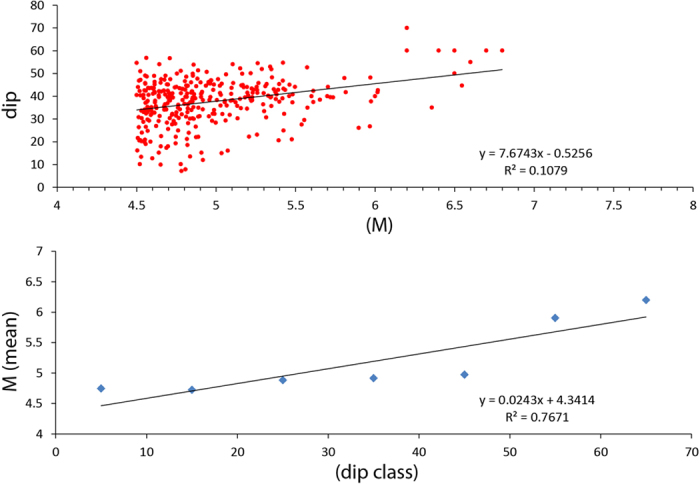
Relationship between normal fault-related earthquakes and fault dip. (**A**) Magnitude frequency distribution for 325 normal-fault earthquakes in the European-Mediterranean region (red dots, time span 1997–2015 and focal depth <40 km) extracted from the RCMT^45^, plus 7 other well constrained historical events. The preferred dipping plane of each focal mechanism is selected on the basis of regional tectonic considerations, and plotted as a function of the magnitude Mw. A magnitude of Mw 4.5 is considered the lowest magnitude of completeness. The regression line shows increasing M with dip, whereas the frequency of the events decreases. (**B**) Magnitude mean value of the Mw >4.5 normal fault earthquakes is plotted as a function of the fault dip angles binned by classes every 10°. Again, the magnitude increases with dip, with a significant correlation R=0.88 (Fisher and Yates, 1963).

Along a normal fault, the hangingwall moves downward (falls) at the coseismic stage. As a consequence, gravity contributes to the generation of the motion, thus, for a constant falling volume, the larger the vertical movement, the larger will be the gravitational energy released. Following the Mohr-Coulomb criteria, a normal fault ideally nucleates at about 60° ^2^. However, normal faults develop at variable angles as a function of the static friction *μ* (Fig. 3). The higher the friction of rocks, the steeper is the fault; vice versa, the lower the friction, the shallower the fault dip. With a constant extension, the shallower the dip, the lower is the vertical component of the fault slip, as predicted by finite elements models (see below). Therefore low-angle normal faults deliver lower energy than steeper normal faults (Fig. 3). This inference is consistent with the small number and the low magnitude of earthquakes nucleated along low-angle normal faults^33^,^34^,^35^,^23^. Low-angle normal faults (LANF) may creep in case of low friction fault rocks^36^. A decrease in friction (e.g., moving from granites to evaporites) induces a decrease of the vertical component of displacement and a lowering of the gravitational potential energy. Normal friction (*μ* = 0.6–0.8) rocks (e.g., platform carbonates) need higher energy to break and slip than low friction (*μ* = 0.2) rocks (e.g., shales, evaporites). Since low friction rocks may easily slip with lower energy^37^,^38^, in active tectonic settings they tend to be associated with higher strain rates (Fig. 4). Therefore, in the brittle domain, normal faults cross-cutting low friction rocks creep faster than normal faults in high friction rocks, which may be activated only with higher amount of stored energy, thus providing larger magnitude earthquakes (Fig. 4). The volume variation in friction may therefore determine strain rate gradients and stress accumulation, both vertically and laterally. However, the variability of strain rate in active tectonic zones may be related either to the relaxation after past earthquakes^39^,^40^,^41^,^42^, or to lateral variation of the average friction of a given crustal volume. Moving along both strike and dip of the Apennines belt, variations in velocities and consequently in strain rate are observed^41^. These may be related to rock friction gradients generated by the alternation of abundant low-friction shaly or evaporitic layers, enhancing creep, and of high-friction carbonatic rocks. Aseismic creep is frequent along active faults^43^ and may be related to low friction lithologies in the upper brittle crust. This may the case also for stretching along passive continental margins^44^ where listric normal faults detach into salt or shales, dissipating part of the gravitational energy during creep (e.g., Fig. 3). The selection of normal fault-related earthquakes from the European–Mediterranean Regional Centroid Moment Tensor (RCMT) catalogue (http://www.bo.ingv.it/RCMT/45) shows that the magnitude increases with the fault dip (Fig. 5). Moreover, according to the Gutenberg-Richter power law, also the frequency of the events decreases with those parameters. Therefore friction controls fault dip, and fault dip determines the vertical component of the coseismic displacement, plus the depth of the BDT constrains the maximum volume involved. Normal fault maximum magnitude earthquakes develop near the base of the brittle upper crust because they may involve the larger volumes. The higher these parameters, the larger are the earthquake magnitudes.

In addition to friction, other factors control seismicity. It is well known that fluids may further deeply control rock mechanics. Conversely, fluids flow is constrained by the pressure gradients and their modification at the coseismic stage^46^,^47^. Moreover, where heat flow is high, such as along mid-ocean ridges, in spite of high velocity spreading rates like in the East Pacific Rise, the seismicity has generally low magnitude because of the low friction of the thin crustal layer (e.g.,^48^). Moreover, the smaller involved volumes associated with normal faults prevent high magnitude earthquakes^10^.

## Discussion

**Figure 6:**
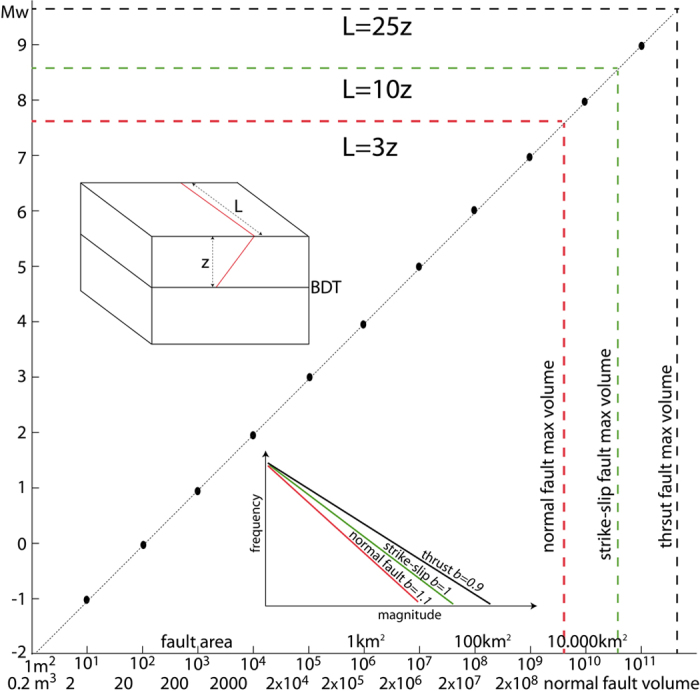
Empirical relationship between earthquake magnitude and fault rupture area. The volume involved by normal faulting is added below. Each type of tectonic setting has its own involved volumes, i.e., determining the maximum expected magnitude. The maximum volumes are computed assuming L = 3z (normal faulting), L = 10z (strike-slip fault) and L = 25z (thrust fault), where L is the fault length and z the involved volume depth. The different types of faulting have also different *b*-value of the Gutenberg-Richter relation^9^, supporting that besides the different involved volumes, earthquakes may have different mechanisms.

Earthquakes are usually considered as having a single elasticity driven mechanism. Earthquakes are elastic waves, but we may discriminate about their source of energy. While transcurrent and contractional tectonic settings transform part of the elastic energy accumulated during the interseismic stage into elastic waves, in extensional tectonic settings the source is gravitational potential energy. We have shown here that earthquakes associated with crustal normal faults may have a different physics and associated with a peculiar phenomenology. For example, based on regional cases, the maximum length of the normal fault activated during a graviquake measured along strike is about three times the hypocentre depth (Fig. 2; Table 1), a value much smaller than those observed in other tectonic settings (Fig. 6; Table 1). Moreover, the seismic volume is laterally bounded by an interseismic conjugate dilated wedge required by the brittle-ductile transition strain partitioning. Therefore the maximum mass of the hangingwall collapsing at the coseismic stage and the available gravitational potential energy that determines the maximum magnitude associated with normal faulting can be computed. A deficit of Mw greater than 1.5 is observed between the radiated seismic energy and the available gravitational potential energy. The difference implies a dissipation of energy in shear heating, heat flow and cataclastic deformation hundreds times larger than the seismic energy, as expected in previous works^5^. Therefore, elastic waves generated at the hypocentre represent the transformation of only a tiny part of the dissipation of the potential gravitational energy by the shock as predicted by previous studies^5^,^17^.

The higher *b*-value (1.1) of the Gutenberg-Richter indicates that normal faults generate lower magnitude events than the remaining fault types. If elasticity were the mechanism determining normal fault seismicity, we should rather expect the same value in all the tectonic settings. In fact elastic potential energy for a spring is the same either in compression or in extension (1/2KX^2^, where X is the spring displacement and K the spring constant), being opposite only the sign in case of stretching or contraction. We rather see that in extensional tectonic settings the involved volume is smaller than in strike-slip and contractional tectonic settings; this implies different parameters and possibly a different mechanism determining the energy dissipation (simply gravitational collapse rather than elastic rebound). Therefore, the seismic radiation should be very peculiar as a function of the energy source. The improved localization and discriminant methods using stochastic sensor networks may help to investigate different types of hypocentres, providing useful information in order to identify seismic events and earthquake types such as graviquakes^49^,^50^,^51^.

## Conclusions

We highlight the following observations: the doubling of the hypocentre depth makes the volume about 8 times bigger; the available gravitational energy is at least 15–20 times larger for the deeper earthquake (Fig. 2). In both cases, the observed magnitude is far lower than the dissipated energy. In terms of energy ratios, the radiated seismic energy is about 1% of the total energy gravitational budget accumulated in the interseismic period and delivered at the coseismic stage (Fig. 2). It has been demonstrated that gravitational energy variations associated with earthquakes are partly radiated out of the source and in part accommodated by frictional dissipation^15^ as documented by the diffused cataclastic deformation and fractures in rocks adjacent to normal fault planes (fault core and damage zone). It has been shown that most of the energy dissipated by the earthquake is transformed into heat^52^. Therefore, the elastic waves generated by an earthquake may represent only 1/100 part of the transformation of gravitational energy delivered by the collapse of the fault hangingwall. Studying pseudotachylites it was concluded that 97–99% of the energy was dissipated as heat during seismic slip^16^. It is beyond the scope of this work to separate and quantify the amount of energy dissipated either by heat flow or fracturing. However, these observations are consistent with the fact that the energy radiated by all earthquakes^53^ is potentially only a minor part of the total energy budget of geodynamics^54^.

Unlike the other tectonic settings, normal fault-related earthquakes occur in favour of gravity. Given the observation that the potential gravitational energy of a volume collapsing during an extensional earthquake is far bigger than the radiated seismic energy, we suggest that 1) the gravitational fall of the hangingwall volume of a normal fault controls extensional earthquakes and that 2) most of the energy is dissipated into shear heating, fracturing, cataclasis, and partly closing the inferred dilated wedge during the interseismic period; moreover, 3) the energy released during an earthquake generated by a normal fault increases with the involved collapsing volume (Fig. 6), the dip of the fault (Fig. 3), and the static friction of involved rocks (Fig. 4). In fact, in the recorded catalogues, the frequency of earthquakes decreases with dip and magnitude (Fig. 5). Larger earthquakes form along steeper normal faults, and the larger the volume, the bigger is the coseismic displacement.

**Figure 7:**
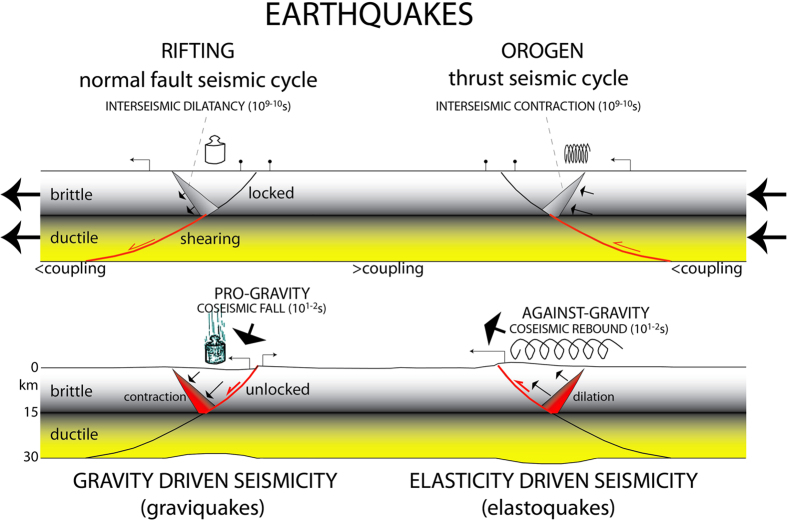
Simplified classification of earthquakes as a function of their energy source. Earthquakes are distinguished depending on whether they are generated by gravity in extensional tectonic settings or by elastic rebound in strike-slip and contractional tectonic environments.

This model of normal-fault earthquake is different from the ideal elastic-rebound model. Being rather dominated by gravity, it could be defined as a “graviquake” (Fig. 7). Since its mechanism is different, we may expect a peculiar evolution and different precursors with respect to the typical strike-slip or thrust-related earthquakes, where the accumulated energy is mostly elastic (“elastoquake”). Graviquakes release potential energy easier than elastoquakes, particularly because they work in favour of gravity. The different mechanism and source of energy of normal-fault earthquakes may explain the smaller involved volumes and magnitude with respect to the other faults generating seismicity. Unlike the elastoquakes that need much more energy to be activated, graviquakes have in fact a smaller ratio (around 3) between fault (and volume) length along strike and hypocentre depth when compared to the other tectonic settings (maximum ratio >10 to about 25 for megathrusts, Table 1).

This model is consistent with earthquakes along intraplate rifts, but it may not entirely apply to normal fault earthquakes generated as aftershocks of thrust faults along the bending of a subducting lower plate or in the hangingwall of a megathrust such as the outer trench-slope faulting associated to the event of 2011 Mw 9.0 off the Pacific coast of Tohoku^55^,^25^; these earthquakes involve large crustal volumes and may therefore generate higher magnitude.

The phenomenology associated with the coseismic stage of graviquakes may be summarized into a number of possible precursors such as: 1) foreshocks in the dilated wedge that formed during the interseismic period, indicating that the overlying volume cannot be sustained anymore; 2) light subsidence of the hangingwall detected by InSAR data; 3) increase of fluids release and increase of pore-pressure at depth demonstrating and catalysing the instability initiation. The achievement of this self-supporting critical state will eventually culminate into the collapse of the hangingwall of the normal fault and the closure of the previously formed conjugate dilated wedge, which may account for a partial dissipation of the abundant available potential energy. The monitoring of these parameters in areas of low strain rate along active faults may eventually provide potential multidisciplinary tools for normal fault-related earthquake prediction. Low magnitude earthquakes in the dilated conjugated wedge of a normal fault may be precursors of the instability of the hangingwall^10^, which will eventually fall.

## Methods

Relating magnitude of earthquakes and faults by means of empirical equations is a common practice in seismological studies. This approach follows the well-known relationships between fault geometry (fault area, dip and seismogenic depth^56^,^26^), fault kinematics (compressional, extensional or strike slip regimes^57^) and maximum energy releasable by a selected system. Moreover, it has been noted that the moment magnitude (Mw) may significantly differ from the magnitude energy (Me)^58^. Available empirical relationships link the magnitude of an earthquake with a bi-dimensional system (the fault). However, during an earthquake a three-dimensional portion of the seismogenic crust is affected by coseismic displacement. As we aim to define the maximum seismic potential given by a volume of rock, the starting point is to define the three-dimensional geometry of the system (i.e., length, depth and width; hereafter brittle volume). Each type of tectonic setting has its own involved volumes, i.e., determining the maximum expected magnitude. The maximum volumes are computed assuming *L *=* a∙z* where *L* is the fault length, *z* the hypocentre depth and *a* is a parameter that varies as a function of the tectonic setting. A shape ratio of *L *=* 3z* (with L and z lateral extent and height of the brittle volume respectively) is consistent with literature data in extensional tectonics^26^,^59^. Therefore maximum *a* is ~3 for normal faulting, reaches ~10 for strike-slip fault and may be larger than 25 for thrust fault (Table 1). The larger the involved volume, the higher is the expected magnitude. Normal faulting has the smaller maximum volume with respect to the other tectonic settings and the maximum potential magnitude is around 7.5–7.7, both theoretically and observed (Fig. 6). Earthquakes close to this magnitude occurred in several extensional environments, regardless the stretching measured by GPS, e.g., both in areas with some mm∙yr^−1^ extension, up to few cm∙yr^−1^. This implies that the involved volume primarily controls the earthquake magnitude, whereas the rate of extension determines the recurrence time of the earthquakes, with shorter recurrence for faster speeds.

Therefore, defining the brittle volume depends on fault dip, brittle-ductile transition depth, rupture length and tectonic setting. In extensional environments, a fixed normal fault dip angle of 45° or 60° (according to average values^46^) is used. We may also infer an empirical relationship between earthquake magnitude and fault rupture area, and from the fault rupture the involved volume (Fig. 6). Regardless the tectonic setting, fault rupture area increases by about one order of magnitude for each magnitude increment^60^,^61^.

In order to quantify the energy along a normal fault, we first compute the volume in the hangingwall of the normal fault (e.g., assuming the BDT as the hypocentre depth, the fault dip, the conjugate wedge dip). The mass is the volume multiplied by the density (e.g., 2.5–2.7 g∙cm^−3^). Then from the mass we can quantify the gravitational potential energy in joule assuming a given vertical component of the fault throw (kg∙m^2^∙s^−2^), which can be transformed into the available energy at the coseismic collapse. However, the resulting magnitude is far larger than the observed one and the computation of the real magnitude from the volume potential energy can only occur with a correction radiation coefficient of 0.02. As examples, we calculated the energy released by three prismatic volumes above hypocentres respectively at 7, 14 and 21 km depth (Fig. 2). The first two may be the Italian earthquakes 1997–1998, Mw 5.7–6 Northern Apennines seismic sequence nucleated at about 7 km depth^62^, and the 1980, Mw 6.9 Irpinia event having the mainshock at about 14–15 km^27^. If the faults dip 45° and ruptures cross-cut the entire overlying rocks, assuming a conjugate wedge at about 60°, emerging at the surface at a distance of 11 and 22 km from the seismogenic faults respectively, the involved volumes amount to ca. 808 and 6468 km^3^. The hypocentre depth, the distribution of the seismicity in the fault hangingwall and InSAR data of subsidence may quantify the volume of other future regional examples. With 0.5 m displacement and with an average density of 2600 kg∙m^−3^, the gravitational energy delivered by the 1997–1998, Mw 5.7–6 Northern Apennines event is around 7.3∙10^15^ J. This amount of energy would correspond to an Mw 7.6 earthquake, far greater than what is observed in nature along faults having such parameters, i.e., Mw 5.8–6.1 (Fig. 5). This means that gravity alone is far more than enough to generate normal faults earthquakes as already observed^14^. Assuming a hypocentre at 14 km depth and a coseismic displacement of 1.5 m, the gravitational energy delivered by the 1980 Irpinia event (that could have been steeper and had even a bigger slip) is instead around 1.7∙10^17^ J. This would mean an event of Me 8.6, far greater than the observed earthquakes that usually are about Mw 6.7–6.9 (Fig. 2). The 1980 Irpinia earthquake occurred along an about 60° steep fault, which would give an even bigger potential gravitational energy. The rupture evolved into 3 shocks, being the first and the second (18 s later) located along the same master NE-dipping normal fault, and the third (at 40 s) along the conjugated inferred antithetic SW-dipping dilated wedge, which evolved into a normal fault. Italy is one of the areas with the strongest normal faulting-related earthquakes, possibly due lithospheric stretching in an area of thick continental crust and larger volumes involved, consistent with graviquake model predictions.

In order to test the graviquake model, finite element dynamic modelling was performed, using the commercial COMSOL Multiphysics 3.5 software (http://www.comsol.com/). In particular we focussed on the coseismic fall of the hangingwall, adopting different fault dip angles. The model adopts 2D plane strain approximation and an elastic rheology (Young’s Modulus: 2∙10^11^ Pa; Poisson’s Ratio: 0.33) and the setup is consistent with previous work^10^. The model geometry is shown in Fig. 3b. The model is 20 km deep and 80 km wide and is separated in two distinct parts by normal faults that in different calculations dip 15°, 30°, 45° and 60° (different colours in Fig. 3b). The finite element grid is made of triangular linear Lagrange elements. Gravity is applied as a body force to all the elements assuming constant density (2850 kg_*_m^–3^) and gravity acceleration (9.81 m_*_s^−2^). The behaviour of the fault is modelled as a contact body (contact pairs in COMSOL’s nomenclature) and is varied (locked or unlocked) in space and time. The unlocked sectors of the fault are modelled as no-friction planes, while locked portions are modelled as identity pairs. The deeper (>10 km) part of the fault is always modelled as unlocked, simulating the ductile (aseismic) slip of faults at depth. A free-horizontal-slip boundary condition (roller in COMSOL) is imposed at the bottom of the model, whilst a free-vertical-slip boundary condition (roller in COMSOL) is imposed at the right side of the model. Free boundary condition is imposed at the model surface.

A displacement of 0.9 m (directed to the left to simulate normal faulting) is imposed in 10 steps to the left boundary of the model, while the seismogenic portion of the fault (shallower than the BDT depth) is kept locked simulating the stress and strain accumulation during the interseismic period of the seismic cycle. The remaining boundary conditions are unvaried. At the 11^th^ step (1 m displacement), the seismogenic fault is unlocked to simulate coseismic slip. Our models reproduce the stress and strain fields described by^10^, not further discussed. As the coseismic fall of the hangingwall is concerned, Fig. 3c shows that the steeper the fault, the shorter is the portion of the hangingwall characterized by coseismic subsidence. In contrast, the steeper the fault, the greater is the hangingwall fall. The maximum values of hangingwall fall occur at the fault and are summarized in Fig. 3d.

## Additional Information

**How to cite this article**: Doglioni, C. *et al.* Normal fault earthquakes or graviquakes. *Sci. Rep.* **5**, 12110; doi: 10.1038/srep12110 (2015).

## Figures and Tables

**Table 1:** Fault length-depth ratio of seismic faults.

Fault type	Earthquake	M	Z (km)	L (km)	L/z	Reference
Normal fault	Pleasant Valley 1915	7.2	15–20	~60	3–4	Wallace *US Geol. Survey Prof. Paper* 1274-A (1984)
Normal fault	Irpinia 1980	6.9	15	~45	3	Bernard & Zollo *J. Geophys. Res.* 94, 1631–1648 (1989)
Normal fault	Corinth 1981	6.4	13	~40	3	Wells & Coppersmith *Bull. Seism. Soc. Am.* 84, 974–1002 (1994)
Normal fault	Edgecumbe 1987	6.5	15	~50	3.3	Beanland *et al. J. Geophys. Res.* 95, 4693–4707 (1990)
Normal fault	L’Aquila 2009	6.3	10	~30	3	Chiaraluce *et al. J. Geophys. Res.* 116, B12311 (2011)
Strike slip	Macquarie Ridge 1989	8.1	12–15	~140	9.3–11.6	Tichelaar & Ruff *Geophys. Res. Lett.* 17, 1001–1004 (1990)
Strike slip	Luzon 1990	7.7	15–20	~150	7.5–10	Velasco *et al. J. Geophys. Res.* 101, 22,419–22,434 (1996)
Strike slip	Landers 1992	7.3	12	~85	7	Massonnet *et al. Nature* 364, 138–142 (1993)
Strike slip	Izmit 1999	7.5	15	~160	10.6	Cakir *et al. Geophys. J. Int.* 155, 93–110 (2003)
Strike slip	Sumatra 2012	8.7	35–40	~400	10–11.4	Wang *et al. Geophys. Res. Lett.* 39, L21307 (2012)
Thrust	Chile 1960	9.5	30–40	~900	22.5–30	Barrientos & Ward *Geophys. J. Int.* 103, 589–598 (1990)
Thrust	Alaska 1964	9.2	30–40	~700–800	17.5–26	Ichinose *et al. J. Geophys. Res.* 112, B07306 (2007)
Thrust	Sumatra 2004	9.3	35–45	~1200	26–34	Lay *et al. Science* 308, 1127–1133 (2005)
Thrust	Maule 2010	8.8	25–30	~500	16.6–20	Moreno *et al. Nature* 467, 198–204 (2010)
Thrust	Tohoku 2011	9.0	30	~650	21.6	Simons *et al. Science* 332, 1421–1425 (2011)

Relationship between fault length (L) and depth (z) of the activated volume during earthquakes associated to few of the most energetic and studied events in the three different tectonic settings during the last century. The ratio increases moving from normal to strike-slip and thrust faults.
